# Beatrix Potter, Author, Naturalist, Mycologist

**DOI:** 10.3201/eid2509.AC2509

**Published:** 2019-09

**Authors:** Byron Breedlove

**Affiliations:** Centers for Disease Control and Prevention, Atlanta, Georgia, USA

**Keywords:** art science connection, emerging infectious diseases, public health, art and medicine, Helen Beatrix Potter, Beatrix Potter, Author, Naturalist, Mycologist, mycology, Agaricus augustus, fungal infections, Candida auris, fungi, molds, mushrooms, yeast, about the cover

**Figure:**
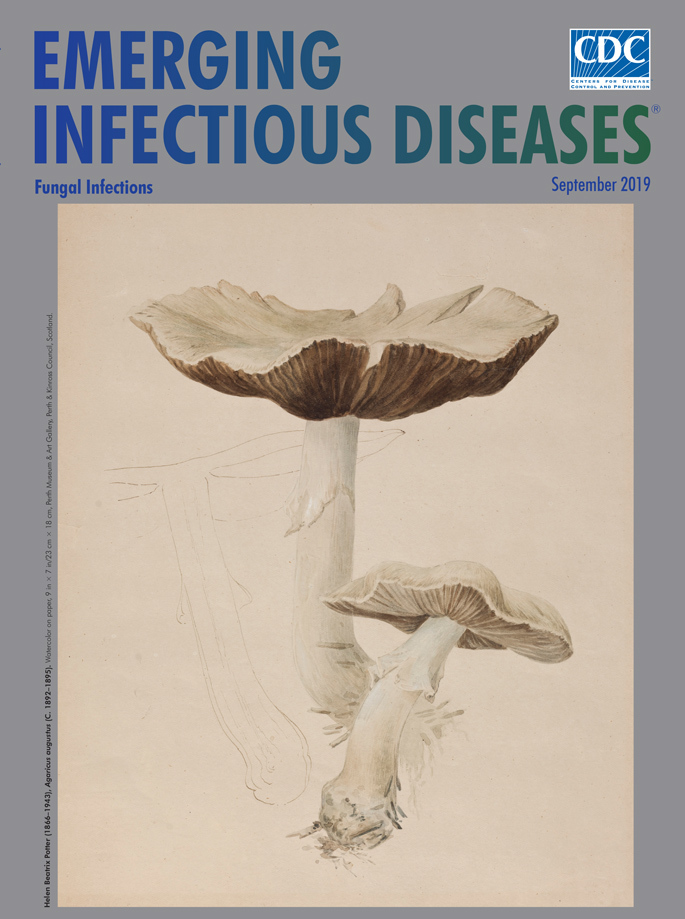
**Helen Beatrix Potter (1866–1943), *Agaricus augustus* (c. 1892–1895).** Watercolor on paper, 9 in × 7 in/23 cm × 18 cm, Perth Museum and Art Gallery, Perth and Kinross Council, Scotland.

Baroque artists often featured mushrooms in still life paintings; medieval artists used toadstools (the name ascribed to poisonous mushrooms) to symbolize evil; and Victorian illustrators often populated fantasy scenes with fungi and fairies. Clearly, fungi have long been a staple of artists and illustrators.[Fn fn1]

Among those was Beatrix Potter. She completed 350 paintings of the various mushrooms and lichen that flourished in the moist green Scottish countryside and English Lake district, locales Potter visited with her family. Before she introduced her anthropomorphic animals such as Jemima Puddleduck, Squirrel Nutkin, and Peter Rabbit in her 23 colorful children’s books that she wrote and illustrated, Potter was an accomplished artist and a naturalist. Biographer Linda Lear writes Potter “never saw art and science as mutually exclusive activities, but recorded what she saw in nature primarily to evoke an aesthetic response.” Potter’s experiences in those lush settings influenced her later interest in land conservation and preservation.

By the early 1890s, notes Lear, Potter’s “interests as an artist and a naturalist had converged on fungi” and she “was drawn to fungi first by their ephemeral fairy qualities and then by the variety of their shape and colour and the challenge they posed to watercolour techniques.” Potter painted her first known watercolors of mushrooms when she was 20 years old. 

During 1892, she befriended Charlie McIntosh, a shy Scottish mail carrier known as the Perthshire naturalist “who used his miles of postal delivery route as a great outdoor laboratory,” according to Marta McDowell, author of *Beatrix Potter’s Gardening Life.* McIntosh, a self-educated naturalist, carefully observed local flora and fauna and encouraged Potter to make her paintings more precise. He sent her specimens, advised her on scientific classification and nomenclature, and instructed her on microscope techniques. As Lear notes, McIntosh “not only provided just the right level of expertise and objectivity to allow Beatrix to advance her skills, but he also gave her the professional validation she longed for.” 

Appearing on this month’s cover is Potter’s sketch showing different stages in the life cycle of *Agaricus augustus* mushrooms. Swedish botanist and mycologist Elias Magnus Fries first identified and named this mushroom in 1838. Considered among the finest of edible fungi, its admirers dubbed it the “Prince of Mushrooms” for its appearance and flavor. 

The Perth Museum and Art Gallery, which houses 25 of Potter’s paintings, describes this watercolor: “The principal image shows it at maturity, where the convex cap evident in the second sketch has flattened out and begun to split round the edges. Similarly, the younger gills appear light in colour but turn a chocolate brown at maturity. . . . At the rear is a large fungus with a broad, shallow, upturned cap. The cap is a creamy white with splits around the edge and dark brown gills beneath. It has a white stem, which is largely straight, curving slightly to the right at the base. In front of it is a smaller fungus of similar shape and colour, except that the cap is not inverted, there are no splits visible at its edges, and the gills are a much lighter shade of brown.” A closer look reveals the outline of another fungus to the left. 

Potter pursued mycology largely on her own and was invited to study fungi at the Royal Botanical Gardens in Kew, London. As Lear discusses, Potter also made microscopic drawings of fungus spores and developed a theory of their germination. In 1897, she submitted a research paper to the Linnean Society of London but was prevented from attending the proceedings or reading her paper because she was a woman. However, Lear writes, “there is no evidence that she had any ambition to be recognized by the scientific community as a mycologist, or that she wished for a life devoted to scientific enquiry,” although Potter’s “watercolours are considered so accurate that modern mycologists refer to them still to identify fungi.” 

The fungi that intrigued Potter include molds, mushrooms, and yeasts. Its members exist in air, water, and soil; experience a range of life cycles; and exhibit an array of morphologic forms. Fungi are integral for decomposition within many ecosystems, have forged symbiotic relationships with other organisms, provide industrial enzymes and metabolites with antimicrobial properties, and serve as experimental organisms. Estimates regarding the biodiversity within the kingdom Fungi suggest that it contains between 2.2 to 3.8 million species.

Some fungi are dangerous and can cause severe health problems, including disability and death. Fortunately, according to researchers Köhler, Casadevall, and Perfect “few among the millions of fungal species fulfill four basic conditions necessary to infect humans: high temperature tolerance, ability to invade the human host, lysis and absorption of human tissue, and resistance to the human immune system.” Fungi that meet those criteria can cause infections on the skin, in the lungs, in the bloodstream; those infections are often challenging to treat, and antifungal resistance is a growing public health concern. An estimated 150 million people have serious fungal diseases, and more than a billion have fungal infections of their hair, nails or skin. 

Diseases caused by fungal pathogens include blastomycosis, coccidioidomycosis, histoplasmosis, Pneumocystis pneumonia, and sporotrichosis. Recent studies estimate that fungal infections, especially those caused by *Candida*, *Cryptococcus*, and *Aspergillus* species, kill more than one million people annually. The concurrent appearance of drug-resistant *Candida auris* on three continents is the first example of a new pathogenic fungi disease emerging from climate change. The urgency underscoring public health efforts to enable earlier detection of fungal infections, apply novel prevention measures, and develop low-toxicity therapies is well founded.

## Footnotes

^1^The North American Mycological Association has a Registry of Mushrooms in Works of Art.
